# Midwives’, obstetricians’, and nurses’ perspectives of humanised care during pregnancy and childbirth for women classified as high risk in high income countries: A mixed methods systematic review

**DOI:** 10.1371/journal.pone.0293007

**Published:** 2023-10-25

**Authors:** Mary Curtin, Margaret Murphy, Eileen Savage, Michelle O’Driscoll, Patricia Leahy-Warren

**Affiliations:** 1 School of Nursing, Midwifery and Health Systems, University College Dublin, Dublin, Ireland; 2 School of Nursing and Midwifery, University College Cork, Cork, Ireland; Trinity College Dublin: The University of Dublin Trinity College, IRELAND

## Abstract

Women classified as ‘high risk’ or ‘complicated’ in pregnancy and childbirth have increased difficulty in accessing humanised care/humanisation in childbirth due to perceptions that this approach rejects the use of intervention and/or technology. Humanised care recognises the psychological and physical needs of women in pregnancy and birth. A mixed methods systematic review using a convergent segregated approach was undertaken using the Joanne Briggs Institute (JBI) methodology. The objective of the review was to identify the presence of humanisation for women with high risk pregnancy and/or childbirth in high income countries. Studies were included if they measured humanisation and/or explored the perspectives of midwives, obstetricians, or nurses on humanisation for women classified as having a high-risk or complicated pregnancy or childbirth in a high income country. Qualitative data were analysed using a meta-aggregative approach and a narrative synthesis was completed for the quantitative data. All studies were assessed for their methodological quality using the MMAT tool. Four databases were searched, and nineteen studies met the inclusion criteria. A total of 1617 participants from nine countries were included. Three qualitative findings were synthesised, and a narrative synthesis of quantitative data was completed. The integration of qualitative and quantitative data identified complimentary findings on: (i) the importance of developing a harmonised relationship with women; (ii) increased time counselling women on their choices; and (iii) fear of professional reputational damage if caring outside of protocols. Negotiating with women outside of protocols may have a wider impact on the professional than first thought. Understanding how healthcare professionals individualise care for women at risk in labour requires further investigation.

## Introduction

The concept of humanisation has had multiple definitions, all of which concur that there is a requirement for women to have a childbirth experience that concentrates not only on the physical outcome of pregnancy and birth, but also positively impacts psychological and emotional outcomes [1–3]. Attributes of humanised practice have been identified as: human interaction, identified by behaviours such as communication, encouragement, and collaboration, which can be summarised as a recognition of the interactive process between the professional and the woman [4]; a benevolence, encompassing traits of the healthcare professional such as patience, tolerance, politeness, caring and strength, and a protagonist; the ability of the woman (or another to enact her wishes) to maintain control over decision making throughout pregnancy and birth [5]. The presence of humanisation was found to result in a reduction of authoritarianism [5]. However, there is less known about healthcare professionals’ (HCPs’) understanding of humanisation. The HCP’s understanding is particularly important if HCPs are to a) advocate for the woman and b) enact a humanised practice. A recent meta-synthesis on the perspectives of HCPs and the humanisation of childbirth suggested ‘invisible boundaries’ may be placed around the women’s choices by HCPs, noted through the use of disempowering language such as to ‘allow’ and ‘let’ [6]. Kuipers [2018] suggests that boundaries are often set by the midwife and the potential for conflict between the woman and the midwife depends on the woman’s and the midwife’s levels of assertiveness. Although HCPs do not see the medicalisation of childbirth in opposition to humanisation and are in agreement that all women can attain humanisation in practice [5,7] the meta-synthesis concluded that HCPs found it difficult to identify humanised practice for women who have been classified as high risk. Physiological birth was found to be integral to humanisation but a safe birth, requiring the use of technology or intervention was of equal importance. This discordance is unsurprising. Historically, humanisation has tended to be a choice for women who chose to opt out of the biomedical model of childbirth and so, it has been used as a method to reduce interventionist practice for women classified as ‘low risk.’ To this end, studies have excluded women classified as high risk or who experience complications [8–12].

In broad terms, a woman is deemed to have a pregnancy or birth classified as ‘high risk’ if a maternal or fetal factor, that may adversely affect the pregnancy outcome, is present [13,14]. Evidence suggests women who have been classified as high risk have difficulty in attaining humanisation [6,8,15]. Women whose pregnancies are classified as high risk are more likely to experience anxiety, negative recall of their experience, and fewer positive experiences [13,16–18]. Unplanned intervention, in particular, may have an adverse impact on the woman’s experiences of childbirth [18,19]. This may be attributed to the woman’s inability to anticipate unplanned pregnancy complications and/or not having an anticipatory roadmap of knowing what to expect [18]. Furthermore, women’s transition to motherhood is also combined with a transition to ‘patient’, an additional adjustment that women who are not classified as high risk may not experience [20]. The label ‘patient’ may also be an unwelcome adjustment for some women who may consider childbirth to be a normal physiological process [20].

Women who are classified as high risk seek to access an individualised and subjective approach to their care. Humanisation is a concept that could potentially provide this approach to maternity care. However, tensions can arise for the woman when decision making is required to maintain a safe birth that results in the need for medical intervention, particularly if they had not planned to do so [21,22]. Women may not be regarded as equals by HCPs in this decision-making process, and some evidence suggests that women cannot be active decision makers if they are classified as high risk [7,23].

There is paucity of research regarding HCPs’ understanding of humanised care, specifically for women with high-risk pregnancy and/or childbirth [2,7,15]. Research in high income countries is also lacking, with most of the research pertaining to humanisation in childbirth having been undertaken in low-middle income countries (LMIC) where the concept of humanisation originated. In light of the seminal work identifying the differing pregnancy and childbirth needs of women globally that coined the phrase *‘too much too soon*, *too little too late’* [24], the needs for HIC and LIMC are different and therefore this work will explore humanisation in HIC only.

Humanisation and its potential to support emotional wellbeing in high-risk pregnancy and birth may be of benefit to women who are already at increased risk of emotional distress. HCPs involved in birth care may have an influence on women’s decisions, and so, there is also a need to understand humanised practice for women classified as ‘high risk’ from the perspective of the professional. A preliminary search of Prospero and the electronic databases was undertaken, and no existing or ongoing reviews of this topic were identified.

We undertook a mixed methods systematic review to identify the presence of humanisation in pregnancy and childbirth for women with high-risk pregnancy and/or childbirth in HIC. The review aimed to answer the following questions:

1. What were the perspectives/views of midwives, obstetricians, and nurses of humanisation in pregnancy and childbirth for women who have been classified as ‘high risk’?
2. What interventions supported humanised care?
3. What tools/instruments were used to examine humanised care in high-risk pregnancy and childbirth?

## Materials and methods

The approach taken was in accordance with Joanna Briggs Institute (JBI) mixed methods review methodology [21]. The protocol for this review was registered with PROSPERO: Registration number CRD42021243286.

### Eligibility criteria

In this study, the understanding of the concept of humanisation, from the perspective of midwives, doctors and nurses who provide care for women in pregnancy and birth, including attributes of humanised care for women whose pregnancy is/was classified as high risk or complicated were included. Studies that identified their population as high risk or complicated were included. The term ‘complicated’ was identified as a term in the literature that encompassed ‘high-risk’ labour and birth and was therefore added to the search strategy terms. Dissertations/theses, systematic reviews, opinion or commentary articles and studies not undertaken in HIC were excluded at this stage.

To meet the objectives of the review, there was no requirement for comparators or control groups. The qualitative component included any study that demonstrated behaviours or attributes of humanisation by midwives, obstetricians and nurses to women who are classified as having a high risk or complicated pregnancy or childbirth in a HIC. The quantitative component included any tools or instruments that measured humanisation in pregnancy and childbirth classified as high risk or complicated in HIC. This review considered all quantitative, qualitative, and mixed methods studies. Studies that were not undertaken in HIC according to World Bank Data [25], that did not allow for extraction of data specific to high risk/complicated pregnancy and birth or that included pregnant women under 18 years old (in order to identify a status of adulthood globally) were excluded. Studies published prior to the year 2000 were excluded following the agreement of an international definition of humanised care [26].

### Search strategy

Due to the lack of literature on humanisation as a concept in high income countries specifically, the terms previously identified as the presence of humanised practice in a recent concept analysis were used to map the attributes and behaviours of humanisation into the search [5] (See Table 1). The search for qualitative and quantitative elements were completed simultaneously on electronic databases CINAHL, Medline, PsychINFO and SocINDEX. The review was in compliance with the Preferred Reporting Items for Systematic Reviews and Meta-analyses (PRISMA) guidelines [27]. The search strategy included Boolean terms “or” and “and” and truncations “*”. Medical subject headings (MeSH) were applied where applicable. The reference lists of studies that met the inclusion criteria were scanned to identify further relevant publications. The search terms used, including truncations were: high-risk or high risk, pregnan*, childbirth or child birth, antenatal or antepartum, labor or labour, intrapartum, human*, interaction*, communicat*, collaborat*, trust*, hope, benevolen*, attitude*, behavio*, optimis*, protagonis*, “self efficacy”, self-efficacy and decision, midwi*, nurs*, obstet*. The major headings were ‘pregnancy, high risk’ and ‘labor complications’, ‘interpersonal relations’, ‘trust’, ‘professional-patient relations’, ‘optimism’, ‘decision making shared’, ‘decision making patient’. The search was completed in February 2022. Ethical approval was not required for this study. See Table 1 for the full search strategy.

**Table 1:** Search strategy–CINAHL plus.

Interface: EBSCOhost research database Advanced search: CINAHL Plus		
#	Query	Results
S1	TI (high-risk OR high risk) OR AB (high-risk OR high risk)	124,659
S2	(MM “Pregnancy, High Risk”)	1391
S3	(MM “Labour Complications”)	2392
S4	S1 OR S2 OR S3	127,582
S5	TI (pregnan* OR child birth OR childbirth OR antenatal OR antepartum OR labor OR labour OR intrapartum) OR AB (pregnan* OR child birth OR childbirth OR antenatal OR antepartum OR labor OR labour OR intrapartum)	216,055
S6	S4 AND S5	10,857
S7	TI (human* OR interaction* OR communicat* OR collaborat* OR trust* OR hope) OR AB (human* OR interaction* OR communicat* OR collaborat* OR trust* OR hope)	707,199
S8	(MM “Interpersonal Relations”) OR (MM “Trust”) OR (MM”Professional Patient Relations”)	44,031
S9	S7 OR S8	737,161
S10	TI (benevolen* OR attitude* OR optimis* OR hope) OR AB (benevolen* OR attitude* OR optimis* OR hope)	456,948
S11	(MM “Optimism”)	1223
S12	S10 OR S11	457,302
S13	TI (protagonis* OR “Self efficacy” OR self-efficacy OR decision*) OR AB (protagonis* OR “Self efficacy” OR self-efficacy OR decision*)	206,195
S14	(MM “Self-efficacy”) OR (MM”Decision Making, shared”) OR (MM “Decision Making, Patient”)	18,728
S15	S13 OR S14	211,599
S16	S9 OR S12 OR S15	1,229,551
S17	S6 AND S16	2719
S18	TI (midwi* OR obstet* OR Nurs*) OR AB (midwi* OR obstet* OR Nurs*)	668,583
S19	S18 AND S19	702
S20	S18 AND S19: Limit Publication year 2000–2022 : Limit English language	615

### Study selection

Following the search, all identified studies were downloaded into Covidence software and duplicates were removed. Title and abstract screening were completed by MC and MO’D. At full text review, all studies were reviewed by MC and the second reviewer was distributed between PLW, ES, MM, MO’D. Any disagreements were resolved through discussion of conflicts that arose.

### Assessment of methodological quality

All studies that met the inclusion criteria were assessed for their methodological quality using the Mixed Methods Appraisal Tool (MMAT) [28]. The MMAT tool can be used for qualitative, quantitative, and mixed methods studies and has been noted for its efficiency, reliability, and content validity [28,29]. There are five questions for each study and a response of ‘Yes’, ‘No’, or ‘Can’t tell’ is required for each question. All studies were assessed for their methodological quality by MC. MM and M’OD shared the second independent assessment for methodological quality. ES adjudicated any disagreements. Regardless of methodological quality, all studies that met the inclusion criteria were included for data extraction and synthesis but a more detailed presentation of methodological quality is addressed in the findings (See S1 File) [30].

### Data collection

A convergent segregated design was undertaken for extraction and synthesis. As the focus of the review was on different dimensions of the phenomena of humanised care this was the most appropriate approach [31]. In accordance with a convergent segregated approach, data were extracted simultaneously pertaining to both qualitative and quantitative data. Quantitative data (including quantitative data from mixed methods studies) and qualitative data (including qualitative data from mixed methods studies) was completed using a template on Google Sheets. Characteristics of all studies were extracted which included specific details regarding the population, context, culture, geographical location, study methods, and the phenomena of interest. The research questions were used to guide the extraction of data. Data extraction was completed by MC and reviewed at regular intervals by the rest of the team.

### Data synthesis and integration

**Fig 1:**
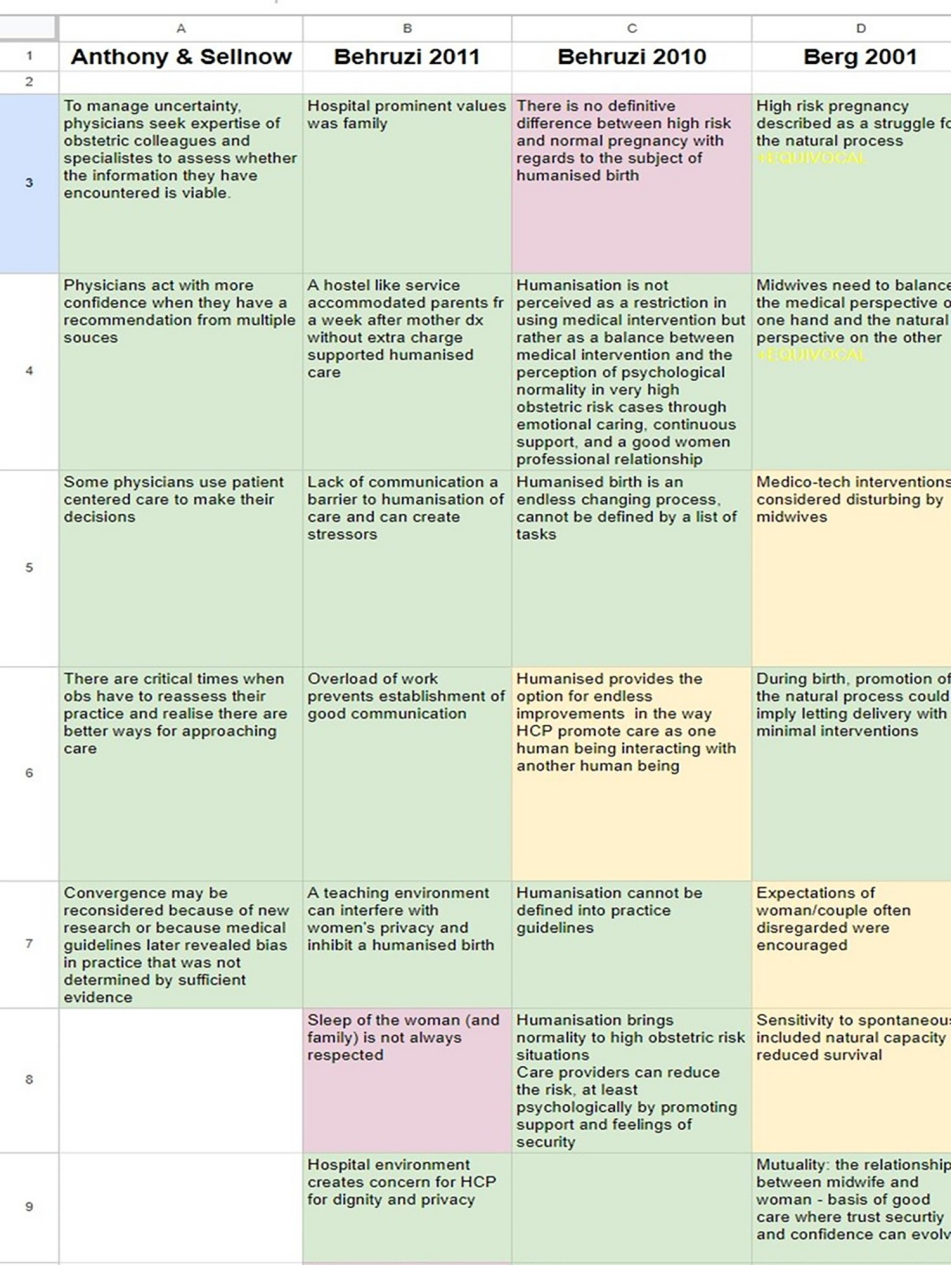
Data extraction and sensitivity analysis.

Following the methodology for a convergent segregated approach, an independent synthesis of qualitative data and quantitative data was completed and then integrated together. Qualitative data synthesis were conducted using a meta-aggregated approach [32]. This approach, grounded in pragmatism places an emphasis on producing findings that inform practice-level theory with a view to making statements rather than a reinterpretation of the data. According to a meta-aggregative approach, three levels of evidence were used; ‘unequivocal’ (where findings were accompanied by data that is beyond reasonable doubt), ‘equivocal’ (where findings were accompanied by data that lacked a clear association with it) and ‘unsupported’ (where findings were not supported by the data presented) (See Table 2). Each study had data extracted onto a Google sheet relevant to the review questions. Each study data was then transcribed onto one sheet and colour coded to visually identify unsupported, equivocal, and unequivocal data (see Fig 1). When complete, categories were formed from the study findings. Synthesised findings were developed from the identified categories. A sensitivity analysis was conducted, through colour coding the levels of evidence to ensure that data identified as ‘unsupported’ did not overly affect the results [33]. Quantitative data synthesis was not possible due to the heterogeneity of the results and therefore a narrative synthesis was completed.

**Table 2:** Definitions of the meta-aggregative levels of evidence.

Level of evidence	Definitions
Unequivocal	Findings accompanied by an illustration that is beyond reasonable doubt and therefore not open to challenge
Equivocal	Findings accompanied by an illustration lacking clear association with it and therefore open to challenge
Not supported	Findings are not supported by the data

The separate qualitative and quantitative synthesis was integrated using a process of configuration. The integration of the separate qualitative and quantitative data does not set out to confirm or refute either qualitative or quantitative data.

## Results

**Fig 2:**
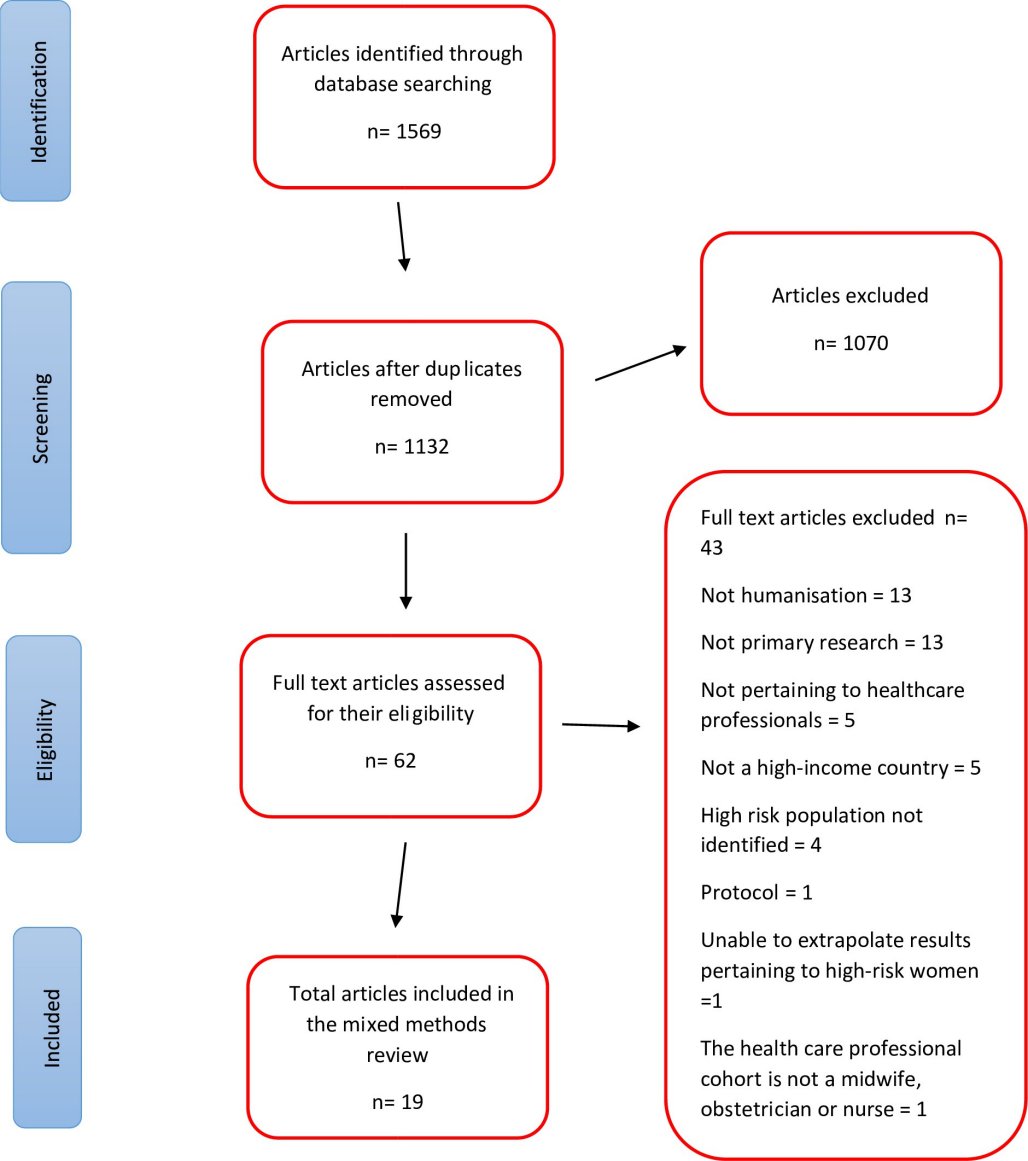
PRISMA diagram.

The search strategy identified a total of 1569 studies. There were 437 duplicates removed which resulted in 1132 studies screened for their title and abstract. A total of 1070 studies were excluded resulting in 62 studies assessed for their eligibility at full text review. There were 43 studies excluded as they were not on humanisation (13), not primary research (13), not pertaining to HCPs’ views (5), not a high income country (5), high risk population not identified in the sample (4), study protocol (1), unable to extrapolate results pertaining to high risk women (1), or in a HCP cohort who were neither a midwife, obstetrician nor nurse in an obstetric field (1). See PRISMA diagram (Fig 2). There were no interventions to support humanisation found in the qualitative or quantitative studies.

### Study characteristics

Nineteen studies met the inclusion criteria and comprised 15 qualitative, 3 quantitative and 1 mixed methods study. Publication dates ranged from 2001–2020 with only one study being published pre 2010 [32]. The included studies were undertaken in ten high income countries with the largest number published from the Netherlands (N = 6) [34–39], followed by Sweden (N = 3) [40–42], the USA (N = 2) [43,44], and the United Kingdom (N = 2) [45,46]. Six countries published one study each; Canada (1), Japan (1), Australia (1), New Zealand (1), Norway (1) and Ireland (1) [7,8,47–50]. See Table 3 for the characteristics of each study. Seven studies identified a participant sample of midwives only [36,37,40,45–47,49]. Two studies recruited a participant sample of obstetricians only [44,48] and one study identified a sample of nurses only [42]. One study described the doctors dealing with an acute obstetric event as physicians as is the norm for the studies country of origin [43]. Nine studies included a mixture of HCPs in their population [7,8,34,38,39,41,43,50]. Three of these studies separated the midwife sample into hospital based, community based or holistic, defined in the literature as midwives who are willing to assist women who refuse recommended hospital care, with a planned homebirth [34,35,39]. Only one study did not separate HCPs in the hospital sample and therefore, midwives and obstetricians were combined as a comparison to the community midwives [35].

**Table 3:** Characteristics of the study.

Author	Purpose	Participants	Methodology
Anthony and Sellnow (2016) [44] USA	To apply the Richie and Spencer framework to medical decision making among obstetricians when managing complexities in pregnancy when multiple options for care exist.	28 obstetricians	Qualitative Interviews Ritchie and Spencer framework analysis technique
Behruzi et al., (2010) [1] Japan	To define professionals’ perceptions related to humanised birth in high-risk pregnancies, and the factors that may facilitate or prevent the provision of humanised care in a high obstetric risk situation.	2 obstetricians 1 professor of healthcare administration 1 academic midwifery professor 3 clinical midwives 2 Focus groups of clinical midwives (group of 3) One focus group of nurse midwifery students (5) and one focus group of midwives in a birthing centre (3)	Qualitative Nine interviews Four focus group interviews Content analysis
Behruzi et al., (2011) [8] Canada	To explore the organizational and cultural factors which act as barrier or facilitators in the provision of humanised obstetrical care in a highly specialised, university-affiliated hospital.	11 professionals from different disciplines. Nurses, obstetricians, paediatricians, and anaesthetists	Qualitative Interviews, field notes, participant observations and a self-administered questionnaire, documents and archives Deductive content analysis
Berg & Dahlberg (2001) [40] Sweden	To describe how midwives experience the care of women during pregnancy, childbirth, and early parenthood when the situation is characterised as high risk from an obstetric point of view or when a complication is manifested for the woman and/or the unborn/newborn baby	10 midwives	Qualitative Interviews Phenomenology
Copeland et al., (2014) [47] Australia	To explore midwives’ perceptions about childbirth and in particular their beliefs about normality and risk and to understand how these perceptions influence clinical decision-making in relation to the use of interventions	12 midwives	Qualitative Interviews Thematic analysis
Engstrom & Lindberg (2013) [42] Sweden	To describe critical care nurses’ experiences of providing nursing care to the new mother and her family after a complicated birth	13 critical care nurses	Qualitative Focus groups Content analysis
Grobman et al., (2010) [43] USA	To understand preferred approaches that HCPs could use when caring for parents who are at risk of giving birth to an extremely premature infant	35 physicians 17 nurses	Qualitative Interviews Analysis not defined
Hilder et al., (2020) [48] New Zealand	To identify key discourse characteristics and patterns that exemplify effective communication practices in consultations in a high-risk antenatal clinic	13 obstetricians	Qualitative Interviews Discourse analysis and thematic analysis
Hollander et al., (2018) [35] Netherlands	To explore experiences of Dutch midwives and gynaecologists with pregnant women who request more, less or no care during pregnancy and/or childbirth	455 community midwives 445 hospital staff (midwife and obstetrician)	Quantitative Anonymous questionnaire Chi Squared analysis: categorical variables. Mann Whitney U tests: Ordinal variables
Hollander et al., (2019) [36] Netherlands	To examine holistic midwives’ motivations and way of practice in order to provide other maternity care professionals with insight into the way they work and to improve professionals’ relationships between all care providers in the field	24 midwives	Qualitative Interviews Grounded theory analysis
Holten et al., (2018) [34] Netherlands	To explore how the wish to birth outside the system was negotiated in consultations/clinical encounters between pregnant women and their health care professionals	5 community midwives 8 holistic midwives 8 obstetricians	Qualitative Interviews Thematic analysis
Offerhaus et al., (2015) [37] Netherlands	To explore the influence of risk perception, policy on routine labour management, and other midwife related factors on intrapartum referral decisions of Dutch midwives	243 primary care midwives	Quantitative Questionnaire (Discrete choice experiment) Linear regression–impact of each factor on referral decisions Pearson Correlation–observed and predicted referral decisions Spearmans Rho–correlation between referral score & percentage of intrapartum referrals
Pieters et al., (2018) [38] Netherlands	To investigate inter-organisational designs for care-cure conditions in which low risk patients are cared for in specialised care organisations and high-risk patients are cared for in specialised cure organisations	Qualitative sample: 7 obstetricians and 19 midwives Quantitative sample: 13 obstetricians and 22 midwives	Mixed Methods Qualitative sample–interviews Quantitative sample–questionnaire Analysis not defined
Risa et al., (2011) [49] Norway	To explore and describe the patterns of verbal communication in antenatal consultations involving encounters between mothers to be and their midwives	6 midwives	Qualitative Recordings of 10 consultations Thematic analysis
Romijn et al., (2016) [39] Netherlands	To gain insight into similarities and differences between midwives and obstetricians in the assessment of a prolonged first stage of labour and the decision to refer a woman to a clinical setting in the Netherlands	Primary care midwives– 69 Obstetricians– 47 Clinical midwives– 31	Quantitative Survey Pearsons’s correlation coefficient Sequential decision model
Sosa et al., (2018) [46] UK	To explore the transition from midwifery one to one support in labour in a midwife-led birth environment to an obstetric-led unit from the perspectives of midwives and women	11 midwives	Qualitative Interviews Thematic analysis
Symon et al., (2010) [45] UK	To examine independent midwives’ management and decision making in 15 instances of perinatal death that occurred at term	14 midwives	Qualitative Interviews Thematic analysis
Wahlberg et al., (2020) [41] Sweden	To explore midwives’ and obstetricians’ experiences, reactions, and interpretations of being part of a severe event on the labour ward	7 midwives 7 obstetricians	Qualitative Interviews Content analysis
Walsh-Gallagher et al., (2013) [50] Ireland	To explore the perceptions of two multi-professional teams in Irish hospitals as to how maternity services to women with disabilities can be improved.	1 ward manager 1 discharge co-ordinator 1 ward sister 6 midwives 1 NNU practice educator CMS PHN	Qualitative Focus groups. Thematic content analysis

There were 1617 participants included in this review in total. A further breakdown of participants can be seen in Table 4. A total of 456 participants were unable to be identified by their profession [8,35].

**Table 4:** Participant breakdown.

Participants by study design		Participants by profession	
Total participants included in the study: 1617			
Quantitative participants	N = 1290	Midwives	N = 969
Qualitative participants	N = 266	Obstetricians (including physicians who provide birth care)	N = 156
Mixed methods participants	N = 61	Nurses	N = 34
		Unable to be identified	N = 456

The data collection methods in the included studies ranged from semi-structured interviews (N = 11) [8,36,38,41,43–48,51], open interview (N = 1), observational (N = 1), focus groups (N = 2), questionnaire (N = 1), discrete choice experiment (N = 1), survey (N = 1), or a combination of both (N = 1). Five studies were analysed using content analysis [7,8,41,42,50] and four studies used thematic analysis [34,43,45,47]. All quantitative studies used descriptive statistics and the quantitative studies used a range of statistical methods including Chi squared analysis [35], Mann Whitney U tests [35], Linear regression [37], Pearson’s correlation [37,39], and Spearman Rho [37].

#### Methodological quality

All quantitative studies were assessed under the quantitative descriptive criteria. There was uncertainty with regards to clarity of the identification of appropriate measurements [35], the representation of the target population [39] and the acknowledgement of the risk for non-response bias [39]. The mixed methods study did not provide clarity for four of the five quality criteria and was methodologically the weakest [38]. Two qualitative studies did not clearly identify a qualitative approach to answer the research questions [45,50] but the remaining 13 studies met all the criteria.

### Qualitative study findings

Studies included a broad range of review questions including questions on communication [34,44,48,49], birth outside the hospital [34,36,37,45,46], experiences of caring for women who have a high risk [7,40,47], and how humanisation is impacted by the organisation [8]. Four studies focused on a specific risk in pregnancy and birth: diabetes [49], recovery after complicated childbirth [42], extreme prematurity [43] and disability [50]. Only two studies identified the concept of humanisation specifically in relation to high-risk pregnancy and birth [7,8]. There were 43 individual findings. Thirty-seven of the findings were unequivocal and 6 findings were equivocal. Three synthesised findings were developed supported by the evidence in the qualitative data. S2 File contains a tabular format of the synthesised findings with the associated evidence.

Synthesised finding 1: Open communication between the healthcare professional and the woman should be encouraged to develop and build a relationship throughout pregnancy and childbirth that identifies both the physical and psychological needs of equal importance as well as the ability for the woman to make informed decisions as an individual or in a shared capacity.

A total of 18 individual findings (and their associated evidence) were extracted from seven of the included studies. Of the 18 findings 13 were unequivocal and 5 were equivocal. (See S2 File).

The attitude and behaviour of the caregiver was noted to directly impact humanised care for women classified as high risk. HCPs recognised that some women experience additional stress in high risk / complicated pregnancy and identified trigger points where women may have increased psychological needs such as at an unexpected admission or in the event the woman and her child are separated [7,42]. Humanisation did not support the separation of the mother from her baby [42].

Humanisation was considered possible through the development of good relationships with women and care and compassion were considered integral to this [7]. Relationships that were perceived as equal [40] were developed through conversation that is considered to be realistic and honest [48]. HCPs understood the importance of successful communication in order to develop a relationship and used verbal and non-verbal communication techniques to build a rapport with women [48]. HCPs appreciated ‘*displays of knowledge*’ and understanding from women regarding their medical condition in their pregnancy including the opportunity to ask questions when with their care provider [48].

Decision making was considered an important aspect of care in humanised practice. However, there was some evidence to suggest an overemphasis was made on the decision making aspect rather than the conversation before a final decision was made [7]. Decision making was considered to be affected by national culture and there were some HCPs who felt that an informed choice in high risk pregnancy was not possible [7]. However, a move away from a paternalistic approach to decision making was considered to be a positive step and in the direction of humanised practice [34]. Midwives acknowledged a difficulty in supporting a woman’s decision making that could increase the risk to the fetus [36]. In particular, midwives were noted to be more likely to want to spend prolonged time periods with a woman [35] and understand women’s feelings, beliefs and opinions [7].

Synthesised finding 2: Inter-Professional relationships and the support they provide are as integral to humanisation in high-risk pregnancy as the relationship between women and the healthcare professional. In particular, collaboration and networking across the multi-disciplinary team is vital. At times, the ideological approach adopted by the professional can impact on the plan of care and cause friction.

A total of 15 findings (and their associated evidence) were extracted from 9 studies. Of the 15 findings, 14 were unequivocal and 1 was equivocal (See S2 File).

For humanised practice, inter-professional team working was integral to ensure women had appropriate care in pregnancy and birth. All HCPs placed their colleagues’ opinions in high esteem, particularly if there was uncertainty in the management of care [44]. However, this also meant that if there were poor outcomes midwives and obstetricians felt scrutinised by their colleagues [41,46]. Midwives experienced self-scrutiny as well at the time of a transfer from one maternity setting to another in case omissions of care would be noted [46]. All HCPs were concerned by reputational damage, but holistic midwives in particular tried to limit this by engaging with hospital services even when supporting women’s wishes to birth at home.

Humanised practice required a network of likeminded caregivers to collaborate together and to support women and midwives identified using their peers as a method to debrief [36,46,52]. A network was seen to improve the quality and accessibility of care [8,53]. However, continuity of carer was still considered important even when women engaged with the multi-disciplinary team [40].

Humanised practice required the provision of consistent information. There was an agreement that there was a need to have a *‘party line’* and get *‘all actors on board’* [41] when speaking to women and their families. Midwives identified themselves as sometimes translating the conversations of medical staff such as obstetricians and neonatologists [43].

Humanised practice did not specify an approach to care. Holistic midwives felt that the burden for adopting a facilitative approach to care was a heavy one [36]. There were no data suggesting a patriarchal approach to care felt a burden. However, some HCPs were unwilling to attend a homebirth if the woman was classified as high risk but were able to empathise with women classified as high risk and why they may wish to birth at home [34].

Synthesised finding 3: Healthcare professionals recognise high risk pregnancy and birth as requiring continued learning and development however the instigation and maintenance of medical intervention must be balanced.

A total of 10 findings (and their associated evidence) were extracted from 6 studies. Of the 10 findings, all 10 were unequivocal (See S2 File).

Midwives acknowledged that good obstetrical knowledge was essential in the care for women classified as high risk [40] and part of the role in high risk pregnancy and childbirth was the recognition and action of a high risk clinical situation emerging [41]. However, the opportunities to develop expertise in a hospital environment were also considered to lead to increased medical intervention [8]. HCPs felt there was reduced opportunity for negotiations and concessions in the hospital setting which limited the practice of humanisation. Over reliance on protocols and guidelines was not considered humanised and instead the ‘client must come first’ [36]. Although HCPs respected the introduction of guidelines and policy, they were wary that new research would identify a different approach, but they felt more confident when they received a recommendation from multiple sources [44].

Staff valued a model of childbirth that provided the flexibility to use technology and/or intervention [8] but recognised the presence of protocols recommending intervention inadvertently resulted in less chance for an intervention free birth [36]. Midwives found birth more pleasurable when it was not governed by intervention. Obstetricians reported their care being evaluated by clinical outcomes [47]. There was an acknowledgement that midwives and obstetricians were striving for the same goal and so a better balance could be reached when a mutual respect was present [34].

### Quantitative study findings

Due to the heterogeneity of the quantitative results, a narrative synthesis was completed. There were no tools or instruments identified in the review that could be used to measure humanisation in high-risk pregnancy and childbirth. The quantitative data found two narratives in the included studies that was considered to be humanised practice by HCPs. Finding a deviation from the norm and identifying a pregnancy that was high risk or complicated was considered to be humanised. Furthermore, the management of women’s requests were considered to be humanised. The management of requests involved more care or less care, for example, declining an antenatal procedure such as a glucose tolerance test or requesting an elective caesarean section.

#### Assessment and referral of high-risk pregnancy

When considering women classified as high risk or complicated in pregnancy, midwives were more likely to underestimate the outcome of spontaneous vaginal birth and overestimate unfavourable outcomes [37]. Identifying deviations from the norm was humanised practice but differed between professional groups. Clinical findings were used to recognise deviations from the norm. The identification and assessment of prolonged first stage of labour was significantly different between professional groups [39]. Primary care midwives (PCM) were more likely to identify prolonged first stage of labour through assessment of cervical dilation, women’s state of mind and the intensity of contractions compared to obstetricians and hospital based midwives [39]. This is comparable to Offerhaus, Otten [37] who also identified that PCMs’ decision making differed from peers and was more impacted by the application of the fetal skull to the cervix and the descent of the fetal skull in the pelvis. PCM were the only professional group to review the woman’s state of mind as part of their assessment. All professionals used cervical dilatation in their decision making but obstetricians attached the most importance to cervical dilatation compared to PCM or hospital-based midwives [39]. The influence of physiological factors such as the intensity of contractions on the decision to refer a woman was significantly lower for hospital-based midwives in comparison to PCMs and obstetricians, who found similarity for this factor [39]. Furthermore, the presence of an active management policy resulted in an increased likelihood that a midwife would refer women to an obstetrician and commence a high risk pathway, although this was not found to be statistically significant [37]. A higher referral score was also found for midwives who worked in areas of reduced urbanisation which may be explained by the addition of travel time into their planning of care [37]. A number of characteristics (gestational age, preferred birth location, partner support, coping skills and language barriers) were found to have less impact on a HCPs’ assessment and decision making than Body Mass Index which was the strongest characteristic for onward referral focusing on the clinical findings [37]. Romijn, Muijtjens [39] found that hospital midwives in particular were more likely for Body Mass Index to affect their decision making for onward referral than PCM or obstetricians. The lack of influence of women’s preferences suggests that clinical factors were the predominant factor in decision making.

#### Managing requests from women classified as high risk

Requests for ‘less care’ were more frequently rejected by HCPs than requests for ‘more care’ [35]. ‘Less care’ was defined as women declining elements of protocolised care. Community midwives and hospital-based staff (midwives and doctors working within obstetrics) had comparable requests for less care in high-risk pregnancy (community midwives 88.9% Vs Hospital staff 83.5%). However, although the quantity of requests was similar, the type of requests differed. Community midwives were more likely to be involved in decision making for less care in diabetes testing whilst hospital staff were more likely to be involved in decision making for less fetal monitoring, assisted vaginal birth and caesarean section [35]. Referrals were more likely to be in the direction of community midwifery to hospital staff and community midwives were more likely to decline requests for less care by women experiencing a high risk pregnancy [35]. However, if a request was declined, a referral to a colleague was possible and community midwives had availed of this option more than hospital staff. This was found to be a statistically significant difference (p = 0.001). All HCPs reported increased time that was needed to counsel women requesting either more or less care of up to 60 minutes in order to provide humanised care. The attitude of the midwife and their willingness to tolerate uncertainty, particularly in the intrapartum stage may impact on referral and management of high risk pregnancy [35,37]. However, holistic midwives were more likely to provide care outside guidelines and protocols and therefore, more likely to experience homebirth for women classified for high-risk pregnancy [35].

#### Integration of qualitative and quantitative evidence

The integration of qualitative and quantitative data was completed using the questions provided by the JBI methodological approach [54] (see Table 5). No interventions or tools to measure humanisation in high-risk pregnancy and birth were identified in this review.

**Table 5:** Integration questions.

Integration questions	
1	Are the results/findings from individual syntheses supportive or contradictory?
2	Does the qualitative evidence explain why the intervention is or is not effective?
3	Does the qualitative evidence help explain differences in the direction and size of effect across the included quantitative studies?
4	Which aspects of the quantitative evidence are or are not explored in the qualitative studies?
5	Which aspects of the qualitative evidence are or are not tested in the quantitative evidence.

**Fig 3:**
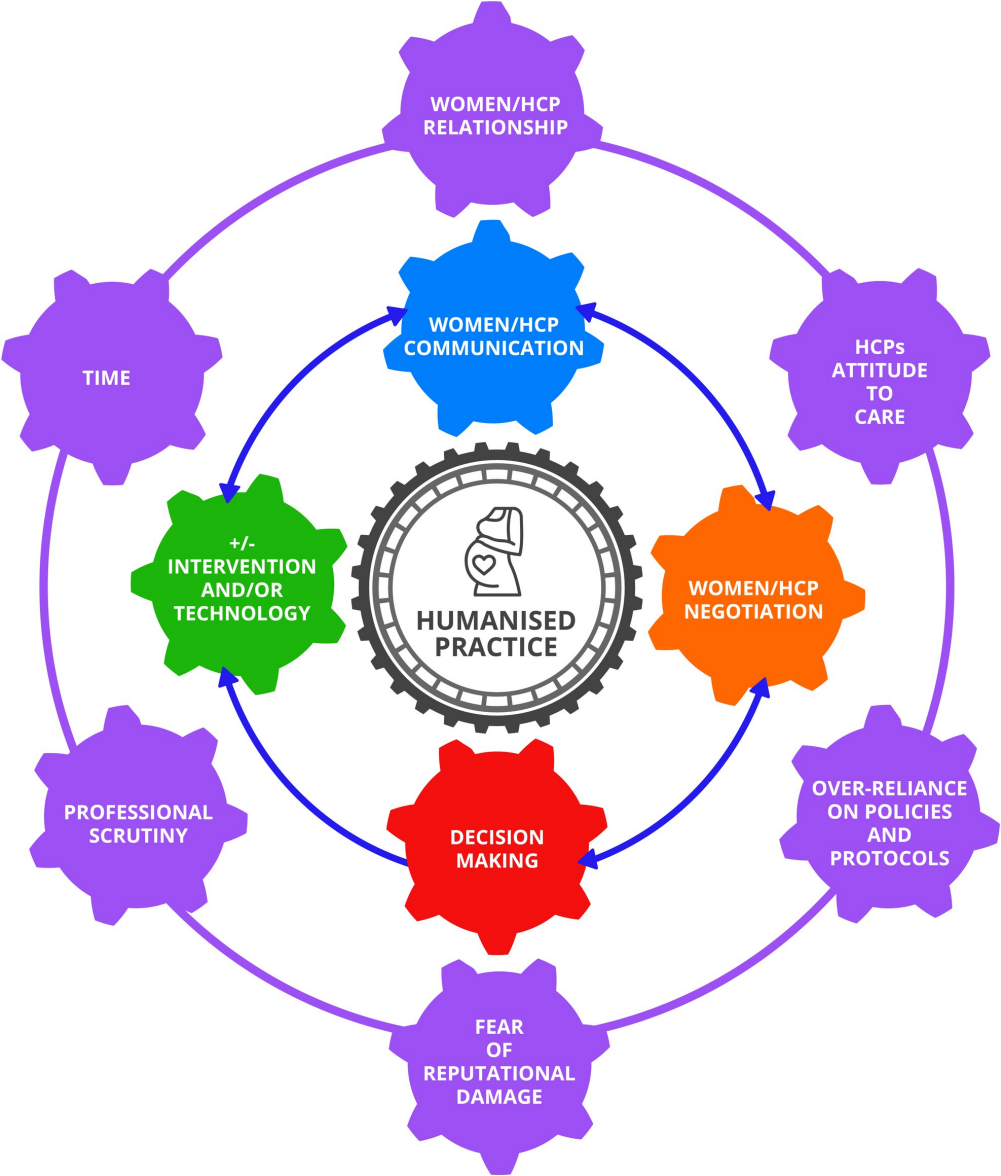
The integrated findings of the review.

Fig 3 illustrates the findings of this review. The inner circle identifies the process of humanised practice for women who are high risk from the perspective of HCPs. Although the inner circle is mostly bi-directional, the relationship between decision making and intervention is one way only. In this scenario, following an intervention, communication must be recommenced. The outer circle contains the barriers and facilitators for humanised practice. The barriers and facilitators are moveable and, depending on where and when they appear can act as a barrier or a facilitator.

There was some alignment in the findings of qualitative and quantitative data. The qualitative evidence acknowledged the importance of the development of a relationship between the woman and the HCP. Continuity of caregiver was provided as a route to ensure the development of a rapport with women, and this also provided an engagement that supported communication, negotiation and decision making prior to a plan of care that may include intervention and/or technology (see Fig 3). The quantitative evidence recognised that humanised practice required increased counselling time at appointments. However, practising in a humanised manner and inviting the woman’s opinions for her care may be detrimental to care systems that rely on seeing a larger group of women, such as in an antenatal clinic setting in a hospital. A lack of familiarity with the HCP may reduce the opportunity for the communication, negotiation and decisions making steps before the plan of care is decided.

Qualitative data showed that HCPs understand the importance of the woman’s right to make decisions for their care. Qualitative data also suggested that women have few opportunities to negotiate their care and doing so may result in conflict. The quantitative evidence suggests that HCPs use the clinical findings as the leading measure to support women’s decision-making process. Only the holistic midwives in the qualitative data recognised the woman’s state of mind in the decision-making process.

There was a strong need for the HCP to feel that they were supported by their colleagues and held in positive regard for their expertise. Quantitative evidence suggested professional scrutiny and the fear of reputational damage was strong enough that at times it was prioritised over the woman’s wishes for her care. To this end, the qualitative evidence suggested that the attitude of the HCP was also important for how care was provided in labour and birth. The quantitative evidence suggests the use of policies and protocols and the importance placed on them by the HCP, (either because of their own belief system or because of a fear of professional scrutiny and potential reputational damage) may be the trigger to how care is provided and proceeds. This may go some way to explaining the direction that care proceeds in times of intrapartum uncertainty. A labour that neither follows physiological norms nor can be defined as pathological may result in two opposing approaches being considered appropriate management of care. Therefore, the HCP may be a deciding factor in the route that is taken. The quantitative evidence identifying midwives as being more likely to overestimate unfavourable outcomes and underestimate spontaneous vaginal birth may be of some credence and lends itself to the argument that the identification of a complication is considered to be humanised practice and so is overemphasised. The qualitative synthesis highlights the importance of the environment and the chance of intervention occurring appears to be more likely in a highly medicalised setting. The combination of these two factors will result in a momentum which may increase the overall intervention rates for birth.

## Discussion

This systematic review of 19 studies is the first to investigate humanisation in high risk pregnancy and birth from the perspectives of midwives, obstetricians, and nurses. There were no tools identified to independently assess humanised practice, so it could be argued that the practice is subjective to the opinion of the HCP. The review highlighted instances where the woman’s voice and her choices may not be prioritized once risk categorisation has been completed and the woman is classified as ‘high risk’. Conversely, although all studies self-identified their participant sample as high risk, none explained the tool or standard used to categorise women as high risk in the first instance. The literature has previously identified midwives having difficulty describing measuring normality often explaining it in the absence of risk factors instead [55]. One reason proposed for this is due to HCPs working within a system of surveillance that is designed to identify and manage hazards [56].

Evidence suggests that HCPs may use their own judgement when deciding the woman’s involvement in decision-making of her own care [57] implying the HCP may proceed with an approach to decision making that has not been explicitly discussed with the woman. Kuipers, Thomson [4] has suggested that there is a ‘relational autonomy’ in which the woman’s autonomy is dependent on her relationship with the HCP. Furthermore, a woman’s autonomy may be impacted by their trust in the professional. Trust may override a woman’s autonomy and the ‘right decision’ is confirmed if women trust their carer, even if the decision is not in line with their original wishes. The HCP’s ability to cope with intrapartum uncertainty may impact the woman’s ability to access her preferred approach. This supports previous literature that HCPs may control the boundaries of choices available to women [6]. Controlling access and/or refusing to provide care to women is not humanised practise as it limits the control that women have over their own care plans.

HCPs over-reliance on policies and protocols did not lend itself to humanised care. An over reliance may support institutional momentum resulting in the protocol or guideline being prioritised above the woman’s voice [58]. Good obstetrical knowledge was seen to minimise an over reliance. Contrary to the HCP’s understanding of humanised practice in this review, the lack of time required to counsel women adequately in complex scenarios may result in reduced opportunities for negotiation and more emphasis on decision-making. Regardless of whether the woman chooses to negotiate care outside of the normal guidelines and protocols, continuity of carer for women who are high risk would support the development of a relationship which may support the counselling, negotiation and the decision-making process identified in Fig 3. Wider literature has also previously identified the importance of building and maintaining a relationship with a known caregiver [57,59] and is now identified as a route to achieving a harmonised relationship with women and a tenet of a women centered ethics approach [60]. A recently published integrative review supports the approach of continuity of carer for all women, regardless of risk status [61]. In contrast to the midwifery participants, obstetricians in this review did not perceive themselves to be measured through the provision of humanised care but instead felt they were measured heavily on survival and quantitative outcomes only. HCPs may benefit from being viewed through the same lens when understanding what humanised practice looks like and how it is measured which may support the promotion of a mutual respect and teamwork which has been found lacking in recent maternity reports [62,63]. ‘Reciprocity’ is a term previously identified in the midwife-mother relationship [59]. In this review, one study used the term ‘mutuality’ [61]. Although similar terms, mutuality and reciprocity have only been researched in a sample of midwives and could benefit from being investigated in both the midwifery and obstetric professions. [40,59]. The presence of reciprocity or mutuality may support humanised practice and reduce the psychological impact of unplanned intervention on women when there is a changing landscape and women transition from maternity setting to another.

Several studies in the review divided midwifery participants into three separate professional groups; the clinical midwife (hospital midwife), the primary care midwife (community midwife), and the holistic midwife (midwives who are willing to assist women who refuse recommended hospital or community care, with a planned homebirth). This division is unusual considering midwives are one professional group, with one scope of practice, a consistent professional philosophy, and on one register with a national regulator. The midwives’ ability to work in several environments may affect their ability to enact that philosophy and alter the subjective perception of humanised care–particularly in the absence of any tool that measures humanisation. Furthermore, the setting for care in pregnancy and birth may affect their understanding of words such as ‘autonomy,’ ‘empowerment’ and ‘decision-making’.

The overemphasis of unfavourable outcomes and an underestimation of the prediction of spontaneous vaginal birth by HCP may inadvertently lead to increasing interventionist practice under the guise of a humanised approach to high-risk maternity care. This is despite evidence-based research to the contrary. Further research is required to understand how HCPs practice individualised care for women at risk in labour and the impact of interprofessional relationships on decision making, particularly for women who wish to negotiate individualised care once classified as high risk.

### Strengths and limitations

A key strength of this review was the robust search strategy and systematic approach to identifying studies eligible for inclusion which was completed independently by two researchers. The use of qualitative and quantitative data strengthens the review, and the use of the convergent segregated approach ensures transparency of the qualitative and quantitative data prior to the integration. However, a limitation of the review is that there was insufficient evidence for a meta- analysis and the review was limited by English only. Studies were not removed based on their methodological quality, but it is noted that the studies of stronger methodological quality contributed more insights into humanisation to the findings. Lastly, this review restricted inclusion to high income countries which could be considered a limitation, but it is also the first study of its kinds to represent humanisation in a high-risk population of childbearing women in high income countries.

## Conclusion

HCPs continue to recognise the presence of humanisation in pregnancy and childbirth but their ability to enact humanised practice for women who are classified as high risk may be reduced when staffing and workload does not meet the needs of the number of women who are in their care. The hospital setting in particular results in difficulties attaining humanisation. The identification of a complication being identified as humanised practice may encourage increased interventionist practice and the clinical environment, but the availability of an intervention must be balanced with its initiation. There does not appear to be an instrument that examines humanised care for women who are high risk, which may contribute to the discrepancies in HCPs understanding of humanisation for women classified as ‘high risk’. Humanisation requires the establishment of a relationship and time, and a continuity of carer promotes both. Negotiating with women outside of protocols may have a wider impact on the professional such as a fear of reputational damage if the outcome is not positive. Further research is required to explore how HCPs individualise care for women who are high risk in labour in a hospital setting.

## Supporting information

S1 Checklist PRISMA 2020 checklist. (DOCX)

S1 File Quality appraisal. (DOCX)

S2 File Synthesised findings with associated evidence of qualitative data. (DOCX)
