# Supplementary material for: Midwives’, obstetricians’, and nurses’ perspectives of humanised care during pregnancy and childbirth for women classified as high risk in high income countries: A mixed methods systematic review
Source: PLoS One. 2023 Oct 25;18(10):e0293007. doi: 10.1371/journal.pone.0293007 (PMC10599554; doi:10.1371/journal.pone.0293007)
Supplement: S1 File — (DOCX) [file pone.0293007.s002.docx]

| **Qualitative Studies** | **1.1: Is the qualitaitve approach appropriate to answer the research question?** | **1.2: Are the qualitative data collection methods adequate to address the research question?** | **1.3: Are the findings adequately derived from the data?** | **1.4: Is the interpretation of results sufficiently substantiated by data?** | **1.5: Is there coherence between qualitative data sources, collection, analysis and interpretation?** |
| --- | --- | --- | --- | --- | --- |
| Anthony and Sellnow (2016) | Yes | Yes | Yes | Yes | Yes |
| Behruzi et al., (2010) | Yes | Yes | Yes | Yes | Yes |
| Behruzi et al., (2011) | Yes | Yes | Yes | Yes | Yes |
| Berg & Dahlberg (2001) | Yes | Yes | Yes | Yes | Yes |
| Copeland et al., (2014) | Yes | Yes | Yes | Yes | Yes |
| Engstrom & Lindberg (2013) | Yes | Yes | Yes | Yes | Yes |
| Grobman et al., (2010) | Yes | Yes | Yes | Yes | Yes |
| Hilder et al., (2020) | Yes | Yes | Yes | Yes | Yes |
| Hollander et al., (2019) | Yes | Yes | Yes | Yes | Yes |
| Holten et al., (2018) | Yes | Yes | Yes | Yes | Yes |
| Risa et al., (2011) | Yes | Yes | Yes | Yes | Yes |
| Sosa et al., (2018) | Yes | Yes | Yes | Yes | Yes |
| Symon et al., (2010) | No | Yes | Yes | Yes | Yes |
| Wahlberg et al., (2020) | Yes | Yes | Yes | Yes | Yes |
| Walsh-Gallagher et al., (2013) | Can’t Tell | Yes | Yes | Yes | Yes |
| **Quantitative Decriptive studies** | **4.1: Is the sampling strategy relevant to address the research question?** | **4.2: Is the sample representative of the target population?** | **4.3: Are the measurements appropriate?** | **4.4: Is the risk of nonresponse bias low?** | **4.5: Is the statistical analysis appropriate to answer the research question?** |
| Hollander et al., (2018) | Yes | Yes | Can’t Tell | Yes | Yes |
| Offerhaus et al., (2015) | Yes | Yes | Yes | Yes | Yes |
| Romijn et al., (2016) | Yes | Can’t Tell | Yes | Can’t Tell | Yes |
| **Mixed Methods study** | **5.1: Is there an adequate rationale for using a mixed methods design to address the research question?** | **5.2: Are the different components of the study effectively integrated to answer the research question?** | **5.3: Are the outputs of the integration of qualitative and quantitative components adequately interpreted?** | **5.4: Are divergences and inconsistencies between quantitative and qualitative results adequately addressed?** | **5.5: Do the different components of the study adhere to the quality criteria of each tradition of the methods involved?** |
| Pieters et al., (2018) | Yes | Can’t Tell | Can’t Tell | Can’t Tell | Can’t Tell |
