# Supplementary material for: Midwives’, obstetricians’, and nurses’ perspectives of humanised care during pregnancy and childbirth for women classified as high risk in high income countries: A mixed methods systematic review
Source: PLoS One. 2023 Oct 25;18(10):e0293007. doi: 10.1371/journal.pone.0293007 (PMC10599554; doi:10.1371/journal.pone.0293007)
Supplement: S2 File — (DOCX) [file pone.0293007.s003.docx]

Supporting Information 2: Synthesised findings with associated evidence of qualitative data

| **Synthesised finding 1 O**pen communication between the healthcare professional and the woman should be encouraged to develop and build a relationship throughout pregnancy and childbirth that identifies both the physical and psychological needs of equal importance as well as the ability for the woman to make informed decisions as an individual or in a shared capacity | | | |
| --- | --- | --- | --- |
| **Category** | **Finding** | | **Supporting Evidence** |
| 1.1  Healthcare professionals want to support women’s decision making in high-risk pregnancy | - Some HCPs used patient centered care to make their decisions (unequivocal) | | *‘I may go to my patient and say some people say that this is the better way, some people say that this is the better way. What do you think fits better for you?’* Physician 10, pg252 Anthony & Sellnow (2016) |
|  | - HCPs recognised women should make informed decisions (unequivocal) | | *‘We should not choose for the mother what she should know, we just facilitate her choice’* Midwife 11, pg54 Behruzi (2010) |
|  | - HCPs have noted a shift towards a more informed clientele (unequivocal) | | *‘She read a lot then, too. She was busy with all kinds of dissertations trying to weigh the risk for herself.’*  Community Midwife, pg1888, Holten et al (2018) |
|  | - HCPs agreed that the movement away from a paternalistic approach to decision making was a positive development (unequivocal) | | *...we are slowly moving from informed consent to shared decision to informed choice. In which the patient makes and informed choice and that you...that I find myself respecting that more and more’* OB/GYN, pg1888, Holten et al (2018) |
|  | - Women’s ability to make decisions reduces obstetricians’ responsibility for negative outcomes (unequivocal) | | *‘If we share, we eliminate responsibility of our staff...we can share the responsibility and decision making and the difficult outcome...’* Obstetrician, pg 54 Behruzi (2010) |
| 1.2  The attitude of the healthcare professional towards high-risk pregnancy and birth may impact the direction of care. | - Midwives want to be close and accessible to women, no matter how the process develops in an emotional and physical sense (unequivocal) | | *‘I give her my time, show her that I have time for her.*  *I stop and I sit down’* Midwife 5, pg 262, Berg (2001) |
|  | - Care and compassion benefit women who experience a high-risk pregnancy (unequivocal) | | *‘I provide humanised care by staying beside the mother and taking care of them to feel safer’* Midwife, pg 55, Behruzi (2010) |
|  | - | Midwives had a strong desire to facilitate physiological birth even when pregnancy was considered high risk (unequivocal). | *‘We tend to keep the doctors out, we keep them out as much as possible, but you have to be strong, and say, ‘I don’t need you at the moment I will let you know if I do’’* Midwife, pg 128, Copeland (2014) |
| 1.3  Healthcare professionals have a clear understanding of what communication should look like between women and the professional | - | The conversation is realistic and honest (equivocal) | *‘The doctor uses very direct colloquial language to talk about the possibility of the uterus ‘pulling apart’ albeit softening the words by lowering the volume of talk’* Doctor, pg 8, Hilder et al (2020) |
|  | - | The focus should be on the negotiation and not on the final decision (equivocal) | *‘We never say from the first say which way is better for a woman. After we meet and we talk with mother and family we gradually offer our suggestion to them as an option, for example, we say it could be better for you to choose this way. From the first we do not ask the woman her choice or decision. We do not offer any option at the first meeting; we need more time after or before the meeting to suggest good options and to have the women and her family to make a good choice’* Obstetrician, pg 54, Behruzi (2010) |
|  | - | Explanations should be delivered in clearly signalled components (equivocal) | *The doctor explicitly indicates they are about to deliver information... informing them the number of topics to be discussed in the consultation as a way of signposting the stages of the explanation.’* Doctor, pg6, Hilder et al (2020) |
|  | - | HCPs want women to be able to ask questions at their appointment (equivocal) | *‘The doctor, while completing their turn in the face of the patient’s bid for a turn, then gives the floor to the patient, maintaining mutual gaze and nodding as a go-ahead’* Doctor, pg8 Hilder et al (2020) |
| 1.4  Professionals should be able to build and maintain a relationship throughout the pregnancy so that trust can prosper. | - | Humanisation is made possible through good relationships with women (unequivocal) | *‘We should establish a confidence between care provider and patient’* Midwife, pg 55 Behruzi (2010) |
|  | - The term mutuality can be identified as a phrase to recognise the development of an equal relationship (unequivocal) | | *‘It must be mutual...the most important thing is to build a relationship, to build bridges, create reliability and security out of chaos...I must establish a line of communication so that she (woman) can learn to trust and understand me’* Midwife 5, pg 262, Berg (2001) |
|  | - Continuity of care supported a woman’s transition to a different maternity setting when her pregnancy was identified as high risk (unequivocal) | | *‘I didn’t want to leave here (birth centre), because I was like a link between the two worlds... the only point that remained in common between the two worlds’* Midwife, Sosa et al (2018) |
| 1.5  The psychological needs of women in high-risk pregnancy and birth should be balanced with their physical needs. | - Psychological needs are as important as physical needs in a high-risk pregnancy (unequivocal) | | *‘Humanisation is not perceived as a restriction in using medical intervention but rather as a balance between medical intervention and the perception of psychological normality in very high obstetric risk cases through emotional caring, continuous support and a good woman professional relationship.’*  Unidentified, Behruzi (2010) |
|  | - Stress and anxiety were intensified by complication of sudden  hospitalisations (equivocal) | | *‘When a doctor comes into a relaxing environment, a woman might feel a lot of stress and everybody experiences a stressful environment’* Professional 2, pg 56, Behruzi (2010) |
|  | - HCPs recognised the mother and child should not be separated after birth (unequivocal) | | *‘It feels difficult... it’s hard. I think that the mother should be with her child, those who are here (ICU) [sic] cannot bond’* CCN 13, pg255, Engstrom (2013) |
| **Synthesised finding 2:** Inter-professional relationships and the support they provide are integral to humanisation in high-risk pregnancy. In particular, collaboration and networking across the multi-disciplinary team is vital. At times, the approach adopted by the professional can impact on the plan of care and cause friction. | | | |
| 2.1  Professionals supported each other in clinical uncertainty but found scrutiny of their practice difficult. | - | Clinical uncertainty is managed by seeking the expertise of colleagues (unequivocal) | *‘If the data are conflicting, then the next thing I arm myself with is to talk to my colleagues to find out if they have any personal experience or any opinions as*  *I start to formulate my plan’* Physician 20, pg 252.  Anthony & Sellnow (2016) |
|  | - | Midwives were noted to confide in their colleagues as a debrief (unequivocal) | ‘*I talked to my colleagues; I find them a great source.’*  Midwife, Sosa et al (2018) |
|  | - | Midwives who did not work within a hospital system often brought women to the hospital in the antenatal period to ensure there was transparency between themselves and their professional colleagues (unequivocal) | ‘*Because I don’t want to be secretive. I explain that to people too. I won’t do that. We are going to the hospital together and we will have a conversation there. You can explain why you want this. Just so they know, and I also want there to be a record of it so that we are welcome in case of a complication.’*  Midwife 5, pg11, Hollander (2019) |
|  | - | HCPs found difficulty in attaining and maintaining humanisation when there was a transfer of care and/or two maternity settings were involved (unequivocal) | ‘*One for epidural, one for no progress and now on Syntocinon, one was ‘span to death’ (referring to spinning babies programme) and then came over here and delivered. The senior midwife added I think the walk over to the labour ward did it’* Midwife, Sosa et al (2018) |
|  | - | HCPs feared the scrutiny of their practice from their colleagues (unequivocal) | *‘Midwife Megan explained that she was not looking forward to going into the labour ward as she feels if anyone says anything she will burst into tears. Megan looks close to tears’* Midwife, Sosa et al (2018) |
| 2.2  The development of collaborative practice between midwives and obstetricians as well as the wider multidisciplinary team  (e.g., NICU) benefits  professionals in high-risk pregnancy and birth | - | A network of support from likeminded caregivers supported humanisation in high-risk pregnancy and childbirth.  (unequivocal) | *‘This was a very kind doctor. I dropped her name in our little club, like: If you want something different, some time, you can try it there...I was very content with that’* Midwife 13, pg 12, Hollander (2019) |
|  | - | Collaboration between staff in the neonatal unit is of particular importance to midwives and obstetricians (unequivocal) | *‘I can phone the neonatal ward and they might say that the child is in an incubator and right now it looks okay and I can tell the mother and she is secure* |
|  |  | HCPs consider that working within a network has improved quality and accessibility of care for women classified as high risk in pregnancy and childbirth (equivocal) | *for maybe half an hour but then I see she becomes worried’* CCN 6, pg 255, Engstrom (2013) |
|  | - |  | *‘We have patients who prefer to be visited at home.*  *There exists home care for high-risk pregnancies. They like care in their environment and we can provide good quality of care for them’* Nurse, pg 11 (Behruzi 2011) |
|  | - | Consistent information is required for parents and must be done by the whole team (unequivocal) | *‘I think one of the things that really helped me is continuous communication with the neonatal intensive care unit because giving the same kind of information to the patient makes us stronger in how we counsel.’* Obstetrician, pg908 Grobman (2010) |
| 2.3  The belief system of the HCP may impact on the approach to care for women who are classified as high risk. | - | Holistic midwives are used as a last resort and are the only group willing to accommodate women’s wishes in their entirety (unequivocal) | *‘Woman wanted a VBAC in hospital with her own midwife. Consultation ended in conflict and refusal to admit. ‘Well then, we will go do it at home, to make a statement. If she is not welcome in the hospital nor in the birthing center then she will have to stay at home and we can’t let her down’ – eventually this woman found a holistic midwife as a community midwife colleagues would not assist’.* Community Midwife, pg1890, Holten et al (2018) |
|  | - | Some HCPs do not want to attend high risk homebirth and support a woman’s wishes (unequivocal) | *’When I see some women come home traumatized after a hospital delivery, I completely understand that you don’t want to go back the next time. But I do believe that talking it through could help solve matters. If it doesn’t, then everyone has their own responsibility to give birth in another way. Just not with me present’* Community midwife, pg1887, Holten et al (2018) |
|  | - HCPs reported more ‘room’ for physiological birth in a high-risk cohort which includes a clearer provision of time and respect for autonomy (unequivocal) | | ‘*And then for me, it became an activist kind of thing, like: this is very important, that we take a stand for this...that women are entitled to their bodily integrity at all times and can refuse care, but in the meantime should not be denied care’* Midwife 12, pg13, Hollander (2019) |
|  | - Midwives strived for continuity of care (unequivocal) | | *‘Midwives tried to minimise the number of persons around every woman’* Midwife 7, pg 262, Berg (2001) |
|  | - Midwives struggled to maintain the atmosphere they wanted after transfer to a high-risk maternity setting (unequivocal) | | *‘They kept knocking on the door asking what was happening and also they wanted to know about the progress. There I really felt the 1:1 care was disturbed. I felt upset, because I felt it was a really important moment. I couldn’t follow her as I would have done because I was continuously going out; in and out, in and out’* Midwife, Sosa et al (2018) |
|  | - Midwives spoke about using intuition in their practice (unequivocal) | | *‘It has something to do with experience and sensitivity, which I also think has something to do with intuition. It is something that only midwives have, midwife-intuition, midwife sense...I do not think we should be afraid of trusting these feelings. We often feel things long before anyone else notices anything’* Midwife 6, pg 264, Berg (2001) |
| **Synthesised finding 3:** HCPs recognise their need for ongoing education in high-risk pregnancy and birth however the initiation and maintenance of intervention must be balanced. | | | |
| 3.1  Continuing education for professionals in high-risk pregnancy and birth creates security in an everchanging landscape | - Increased obstetrical knowledge gave midwives a stronger feeling of security and safety in their professional role and helped them to avoid pathologisation (unequivocal) | | *‘One would have thought that one would become fixated with the complicated, but it has been rather the opposite. I have raised the limit for what I consider normal’* Midwife 7, pg263, Berg (2001) |
|  |  | -There are critical times when obstetricians have to reassess their practice and realise that there are better ways of approaching care (unequivocal) | *‘But that has certainly happened in the past in medicine where people have gone down the same*  *road by consensus and that road has been wrong’* Physician 28, Pg 253, Anthony & Sellnow (2016) |
|  | - | The opportunities for development of expertise in the hospital environment led to gravitation to medical intervention more quickly (unequivocal) | *‘They are valued for their medical performance, not for the fact they listen to their patients, or because they spend time with them’* Obstetrician, pg 10, Behruzi (2011) |
|  | - | Midwives used reflection to understand and resolve events (unequivocal) | *‘Or just the way the baby seems to get its head stuck. I feel like I am just standing by and watching a drama unfold and can’t stop it’* Midwife, pg 129 Copeland (2014) |
| 3.2  Trying to control the environment is a means to balance the image of intervention but could go further in professional practice. | - | Midwives felt they should balance the medical perspective on one hand and the ‘natural’ perspective on the other (unequivocal) | *‘Focus and see the women more, not just the machines, tests and everything surrounding her’* Midwife 1, pg 261, Berg (2001) |
|  | - | Hiding machines may be perceived as a method to reduce the impact of stress from the environment (unequivocal) | *‘Before, 2000 we had machines everywhere in our unit, but we decided to remove all of them to provide a more natural environment which does not use as many instruments’* Midwife, pg 56, Behruzi (2010) |
|  | - | Midwives found birth more pleasurable when not governed by interventions (unequivocal) | *‘I get fearful that the system is going to intervene and carry her into something that she won’t want and that is not necessary, possibly even harm her’* Midwife, pg 128, Copeland (2014) |
|  | - | Obstetricians found the effectiveness of their care is heavily weighted to survival rates and clinical outcomes (unequivocal) | *‘No one gives us an assessment at the end of the month and asks us to look at ourselves and our patients and see where we have been humane...I’m told you have had so many deliveries and your forceps rate is this and your caesarean section rate is that... the indicators of good performance is always expressed in terms of the number of patients, number of births, number of emergency rooms visits and the number of new cases being visited. This is rarely calculated on a measure of the humanisation of care.’*  Obstetrician, pg 10 Behruzi (2011) |
|  | - | Some HCPs were felt to rely too heavily on protocols and guidelines (unequivocal) | *‘It’s everywhere in all the guidelines... the client comes first. I don’t think the client comes first; I think the guidelines come first. And they are no longer guidelines, they have become laws’* Midwife 20, pg 7, Hollander (2019) |
|  | - | Some HCPs felt there was little or no negotiations in the hospital setting to make concessions to plans of care (unequivocal) | *‘And there was a lady who would have been willing to mostly conform to the guidelines, but the door was slammed in her face’* Midwife 7, pg 8, Hollander (2019) |

255
